# Supplementary material for: Observation of the universal magnetoelectric effect in a 3D topological insulator
Source: Nat Commun. 2017 May 15;8:15197. doi: 10.1038/ncomms15197 (PMC5440727; doi:10.1038/ncomms15197)
Supplement: Supplementary Information — Supplementary Figure 1 [file ncomms15197-s1.pdf]

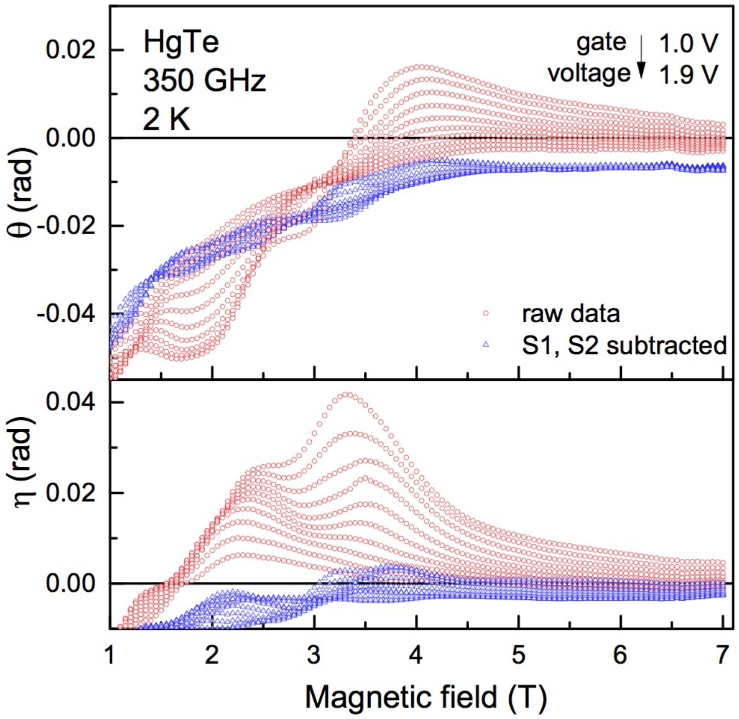

Supplementary Fig. 1: **Subtraction of remaining weak contribution.** Since $\alpha \approx 0.0073 \ll 1$, even very small background contributions may lead to some deviations from the universal value. As demonstrated in Fig. 1 of the main text, a few weaker features are observed in the magneto-optical data in addition to sharp electron resonance. Neglecting the interaction between the subsystems, we may assume that all parts additively contribute to the Faraday conductivity. We start from complex transmission $t_{\rm c}$ and $t_{\rm p}$ data. Using the expression for the transmission through a conducting film on a substrate, the field-dependent transmission is directly inverted to obtain complex conductivities $\sigma_{\rm xx}$ and $\sigma_{\rm xy}$. The transmission data are then fitted assuming Drude-like character of all contributions. Due to additive character of the conductivity, the non-electron contributions may be directly subtracted from $\sigma_{\rm xx}$ and $\sigma_{\rm xy}$. Finally, Faraday rotation and ellipticity are calculated using the formalism described in Methods. We note here that the conductivity associated with Dirac surface states is at least by an order of magnitude higher than that of other states, such that even linear subtraction in rotation angle and ellipticity would be reasonable and would lead to closely similar data. Open circles show the data prior to subtraction and open triangles demonstrate the results of subtracting the non-electronic contributions marked as $s1$ and $s2$ in Fig. 1 of the main text. One can clearly see that all experimental data collapse into single curve, revealing a robust plateau of the Faraday angle above 4 T, which is insensitive to the gate voltage.
